# Brain-wide distribution of reporter expression in five transgenic tetracycline-transactivator mouse lines

**DOI:** 10.1038/sdata.2019.28

**Published:** 2019-02-26

**Authors:** Sveinung Lillehaug, Michael J. Yetman, Maja A. Puchades, Martyna M. Checinska, Heidi Kleven, Joanna L. Jankowsky, Jan G. Bjaalie, Trygve B. Leergaard

**Affiliations:** 1 Department of Molecular Medicine, Institute of Basic Medical Sciences, University of Oslo, Oslo, Norway; 2 Department of Neuroscience, Baylor College of Medicine, Houston, TX, USA; 3 Departments of Molecular and Cellular Biology, Neurology, and Neurosurgery, Huffington Center on Aging, Baylor College of Medicine, Houston, TX, USA

**Keywords:** Gene expression, Neural ageing, Diseases of the nervous system, Animal breeding, Animal disease models

## Abstract

The spatial pattern of transgene expression in tetracycline-controlled mouse models is governed primarily by the driver line used to introduce the tetracycline-controlled transactivator (tTA). Detailed maps showing where each tTA driver activates expression are therefore essential for designing and using tet-regulated models, particularly in brain research where cell type and regional specificity determine the circuits affected by conditional gene expression. We have compiled a comprehensive online repository of serial microscopic images showing brain-wide reporter expression for five commonly used tTA driver lines. We have spatially registered all images to a common three-dimensional mouse brain anatomical reference atlas for direct comparison of spatial distribution across lines. The high-resolution images and associated metadata are shared via the web page of the EU Human Brain Project. Images can be inspected using an interactive viewing tool that includes an optional overlay feature providing anatomical delineations and reference atlas coordinates. Interactive viewing is supplemented by semi-quantitative analyses of expression levels within anatomical subregions for each tTA driver line.

## Background & Summary

**Figure 1:**
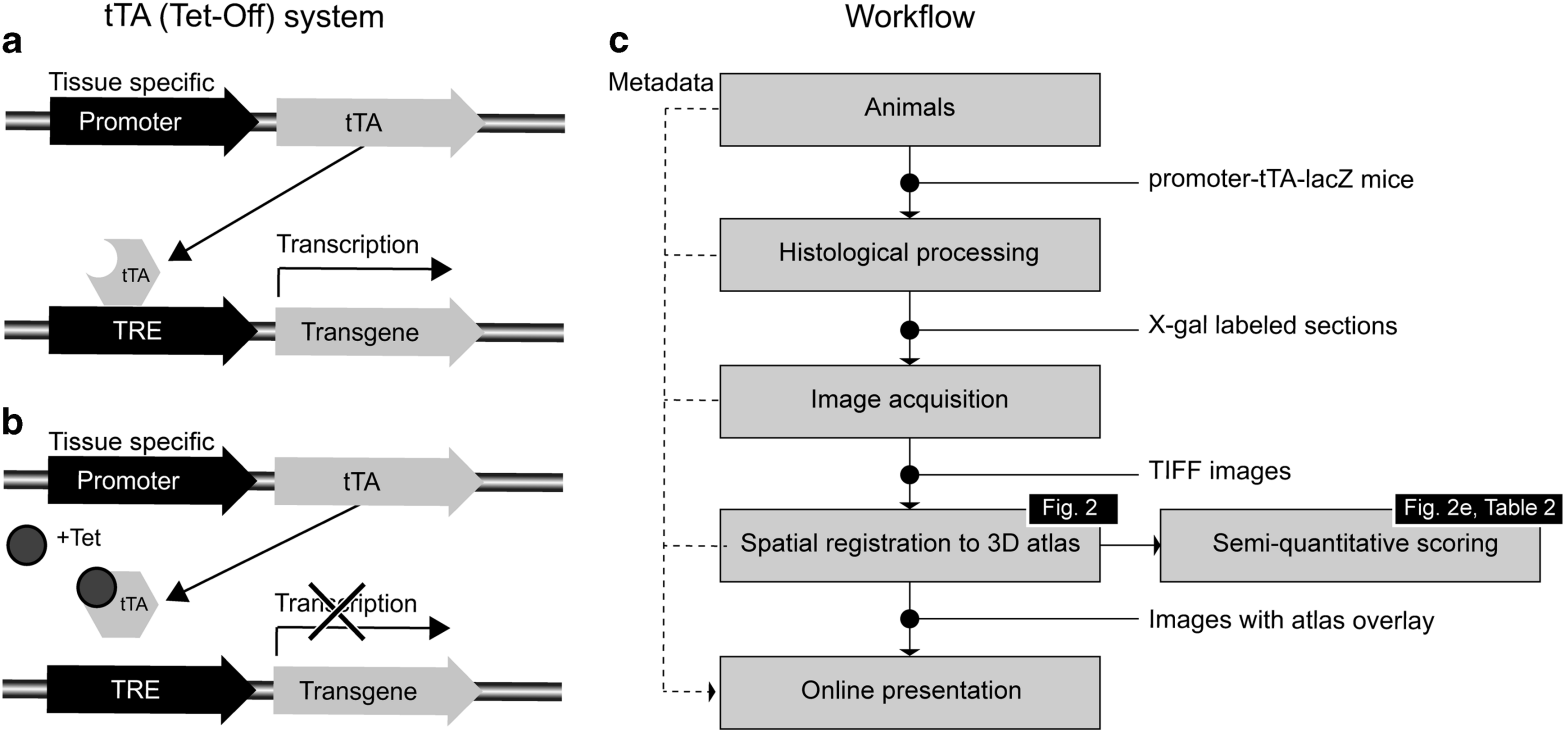
Principle of the tTA/Tet-Off system and workflow. (**a**,**b**) Principle of the tetracycline-dependent regulatory (tTA/Tet-Off) system. A tissue-specific promoter drives spatially restricted expression of the tetracycline transactivator protein (tTA) in the desired tissue. In the absence of tetracycline (Tet), the tTA protein binds to a tetracycline responsive element (TRE) which in turn drives expression of the transgene of interest. (**b**) If tetracycline (Tet) is administered, it prevents tTA from binding to the TRE and activating transgene expression. (**c**) Outline of the workflow used for collection, analysis and presentation of experimental data.

Transgenic mice in which the expression of specific genes can be pharmacologically regulated are powerful tools for studying brain disease^1–10^. One common approach for temporally-regulated transgene expression allows transcription to be exogenously controlled by administration of tetracycline or its chemical derivatives^11–14^. Tetracycline-responsive transcription depends on a bipartite system in which the promoter controlling a transgene of interest is activated exclusively by an artificial fusion protein known as the tetracycline-controlled transactivator (tTA; Fig. 1a,b). The promoter used to restrict tTA expression therefore dictates the spatial distribution of the transgenic protein of interest.

Many different tTA transgenic lines with varying expression patterns have been generated for individual experimental purposes. A database of tet-transgenic rodents lists more than a hundred mouse lines expressing tTA or its tet-inducible counterpart reverse tTA (rtTA), and about one fourth of these are reported to have transgene expression in the brain^14^. Detailed information about the expression pattern of tTA is important for selecting a driver line suitable for the circuit or brain region of interest. Despite this need, comparison of expression patterns reported across the myriad tTA/rtTA lines has historically been difficult because of the varying methods used to locate transgene expression compounded by significant variation in spatial granularity and scope of analyses. Some reports describe brain expression patterns at the level of major brain regions^15–17^, while others focus on selected brain regions with limited documentation of possible expression outside the region of interest^18,19^.

To the best of our knowledge, only four studies have characterized the expression pattern of tTA driver lines in detail across the entire mouse brain. These studies have mapped reporter expression in tTA driver lines based on the promoters for cellular prion protein (Prnp)^4^, neuropeptide Y receptors Y1 and Y5^20^, Ca^2+^/calmodulin-dependent protein kinase IIα (Camk2a)^21^, and neuropsin (Nop)^22^. Three of these studies provide web-based virtual microscopy and repositories with series of histological section images spanning the mouse brain. This information is complemented by semiquantitative analyses of expression levels within selected subregions^4,21,22^ and shared via the Rodent Brain Workbench portal (www.rbwb.org). Detailed anatomical comparison of expression patterns between driver lines nevertheless remains challenging due to differences in the reference atlases used, the criteria for identifying anatomical boundaries, and the plane of sectioning of histological material. Only a subset of corresponding sections can be compared side-by-side, as shown by Odeh *et al.*^21^.

To overcome this limitation in cross-model comparison, we present a repository of serial microscopic images showing lacZ reporter activity in five frequently used tTA-expressing transgenic lines (Prnp; Camk2a; Nop; L7/Purkinje cell protein 2, Pcp2; and Pituitary homeobox 3, Pitx3). The repository serves as a resource for neuroscience researchers working with tet-transgenic models. The image data and associated metadata are made available via the web page of the EU Human Brain Project (www.humanbrainproject.eu)^23^. All images have all been registered to the Allen Mouse Common Coordinate Framework (CCF) version 2^24^. For each tTA transgenic line, the density of lacZ reporter expression across the brain is semi-quantitatively scored. All histological images can be interactively examined online by virtual microscopy with superimposed atlas maps or downloaded for off-line comparison with other experimental materials. The image repository is suitable as a benchmark reference for transgenic mouse models with tetracycline-controlled transgene expression in the brain.

## Methods

### Overview of experimental design

To specify and compare where in the brain conditional gene expression occurs in the most commonly used tTA promoter lines with known brain expression (Prnp, Camk2a, Nop, Pitx3, and Pcp2), we created a repository of microscopic images showing the location of conditional gene expression in five promoter-reporter crosses. In these mice, the transgenic responder line encodes the *lacZ* gene, allowing a β-galactosidase reaction to reveal the spatial distribution of the tTA promoter and thereby indicate where in the brain conditional gene expression occurs. Registration of all histological images to a common mouse brain reference atlas makes it possible to directly compare the regional specificity and amount of expression across the five tTA driver lines. The core workflow is outlined in Fig. 1c.

### Mouse strain generation

All animal procedures were performed in accordance with the National Institutes of Health Guide for the care and use of laboratory animals and approved by local institutional animal welfare committees (associated with Baylor College of Medicine, University of Tübingen, or University of Oslo). The tTA image repository holds image data from 12 adult bigenic mice, representing intercrosses between five promoter-tTA driver lines and two tetO-lacZ reporter lines (Table 1). Experimental details about animal constructs are provided below. Microscopic images from Prnp, Camk2a, and Nop driver lines^4,21,22^ were imported from the Rodent Brain Workbench (www.rbwb.org). Data from single transgenic (tetO-lacZ) control animals, excluding the possibility of endogenous β-galactosidase staining, have been reported earlier^22^. Information on the three strains used to generate Pcp2 and Pitx3 lacZ expression data is provided below.

#### tetO-nls-lac-CMK (tetO-GFP-nls-lacZ-Camk2a 3′UTR)

The lacZ reporter line used for detection of tTA activity in the Pcp2, Nop, and Pitx3 driver lines encoded an in-frame fusion of the *E. coli lacZ* gene with green fluorescent protein (GFP) under control of the first-generation tetO promoter^25^. The reporter construct also included the nuclear localization signal (nls) from the simian virus 40 (SV40) large T antigen and was followed by the three prime untranslated region (3′ UTR) from mouse calcium-calmodulin kinase type IIα (Camk2a). It is unclear from the original report whether the tetO-lacZ-nls-GFP animals were generated on a B6CBA F2 or B6SJL F2 background, but in either case the line was eventually maintained by backcross with C57BL/6 (Dr. Mark Mayford, personal communication). This line was obtained from Dr. Mark Mayford (Department of Neuroscience, Scripps Research Institute, La Jolla, CA, USA) in 2010 and backcrossed to C57BL/6 J for another 4 generations before being mated with the Pcp2-tTA and Pitx3-tTA driver lines as described below.

#### L7/Purkinje cell protein 2 (Pcp2)-tTA/tetO-lacZ-nls-GFP mice (Pcp2-tTA)

Mice expressing tTA under control of the mouse L7/Purkinje-cell protein 2 (Pcp2) promoter were created by Dr. Harry T. Orr (Department of Laboratory Medicine and Pathology, University of Minnesota, USA) and obtained from a colony derived from this stock by Dr. David Nelson (Department of Molecular and Human Genetics, Baylor College of Medicine, USA). The original line was created by injection into FVB/N embryos and maintained on an FVB background. It was received in 2011 and backcrossed to FVB/NJ for another 5 generations before outcrossing to the tetO-lacZ-nls-GFP reporter (see above) for the current studies. The line is available through Jackson Laboratories under strain name FVB-Tg(Pcp2-tTA)3Horr/J, stock #5625.

#### Pituitary homeobox 3(Pitx3)-tTA/tetO-lacZ-nls-GFP mice (Pitx3-tTA)

Pitx3-tTA mice were generated in the laboratory of Dr. Huaibin Cai (Laboratory of Neurogenetics, National Institute of Aging, Bethesda, MD, USA) by targeted insertion of an IRES2-tTA-loxP-neomycin-loxP cassette in exon 4 of the mouse Pitx3 gene^19^. 129/SvJ ES founder males were generated and their offspring were mated with a Cre line to remove the neomycin cassette from the transgene. The line was backcrossed to produce congenic C57BL/6J animals. Animals were obtained from Dr. Mark Mattson (Laboratory of Neurosciences, National Institute of Aging, Baltimore, MD, USA) in 2013 that had been derived from Dr. Huaibin Cai’s original colony at the NIH. The mice were bred at Baylor College of Medicine, Houston, TX, USA for one generation with C57BL/6J before outcrossing the offspring with the tetO-lacZ-nls-GFP reporter line for the current study.

#### Prnp-tTA/tetO-lacZ (Prnp-tTA), Camk2a-tTA/tetO-lacZ (Camk2a-tTA) and Nop-tTA/tetO-lacZ-nls-GFP (Nop-tTA) mice

Description of the construction of Prnp, Camk2a, and Nop-tTA lines are provided in previous publications^4,21,22^. In brief, *Prnp-tTA/tetO-lacZ and Camk2a-tTA/tetO-lacZ mice* were generated by crossing a Prnp promoter line (Prnp-tTA, line F959^25^) or Camk2a promoter line^26^ with a responder line transgenic for a bidirectional reporter gene construct containing the *E. coli* derived *LacZ* reporter gene encoding β-galactosidase^27,28^, and the *Luciferase* gene from *Photinus pyralis* (not used in the present study) (Luc-tetO-lacZ). *Nop-tTA mice* were generated by backcrossing the Nop-tTA mouse line provided by Dr. Mark Mayford (Line tTA-EC^29^; also known as Klk8-tTA; MMRRC strain # 031780) to C57BL/6 J for 1–3 generations before outcrossing to the responder *E. coli* lacZ and green fluorescent protein (GFP) responder line listed above (tetO-lacZ-nls-GFP)^26^.

### Tissue fixation

The procedures described below pertain to tissue from the Pcp2-tTA, and Pitx3-tTA, and tetO-lacZ-nls-GFP mice. The histological procedures used for previously published strains are very similar, but for details see the original publications (Prnp-tTA^4^; Camk2a-tTA^21^; Nop-tTA^22^).

The animals were overdosed with pentobarbital and transcardially perfused with cold phosphate-buffered saline (PBS) containing 10 U/ml of heparin followed by 4% paraformaldehyde (PFA) in PBS. Brains were extracted and post-fixed by immersion for 3 h at 4 °C in 4% PFA/PBS before being cryo-protected by immersion in 30% sucrose/PBS for 48 h at 4 °C.

### Brain dissection and sectioning

After removal of the skull, brains were rinsed in PBS before being incubated for 2 h in PBS containing 5% gelatin at 37 °C (Sigma, G2500) followed by 2 h in 11% gelatin/PBS at 37 °C. Tissue blocks were placed flat-face down onto a 3–4 mm thick layer of solidified 11% gelatin inside a plastic mold and flooded with liquid 11% gelatin solution until the tissue was completely submerged. Blocks were allowed to harden at room temperature for 30–60 min. Gelatin blocks were then released from their molds and immersed in 4% PFA/PBS for 4 h at 4 °C. The blocks were then placed in PBS containing 10% sucrose for 24 h at 4 °C, followed by 30% sucrose/PBS for at least 48 h (and up to 7 d) at 4 °C before they were sectioned.

Cryoprotected gelatin blocks were marked with a razor blade to indicate orientation and placed in a peel-away plastic mold (Polysciences, Inc., 18646A-1) filled with pre-warmed 37 °C PBS containing 3% gelatin. The block was then flash frozen by slowly lowering the mold into isopentane (Sigma, M32631) that had been chilled to −60 °C. After 30 min in isopentane, the block was recovered and the plastic mold removed so that the tissue could be mounted with PBS onto the stage of a freezing-sliding microtome and sectioned at 45 μm. Sections were stored in cryoprotectant (0.1 M phosphate buffer pH 7.4, 30% ethylene glycol, 25% glycerol) at −20 °C until use. Sections were sampled to cover the entire brain; from each brain 32–61 coronal sections or 16–23 horizontal sections were sampled.

### β-galactosidase staining and counterstaining

The *lacZ* gene product (β-galactosidase) was identified using X-gal (5-bromo-4-chloro-3-indolyl-β-D-galactopyranoside) as a substrate. Enzymatic cleavage of X-gal gives rise to an insoluble indigo-blue compound^30,31^. Sections were mounted onto Superfrost Plus slides (Fisher, 12-550-15) from tris-buffered saline (TBS) and air-dried for 48 h before use. Slides were rehydrated in running tap water for 5 min and rinsed for 15 min at room temperature in PBS containing 2 mM MgCl_2_. The slides were then incubated in pre-warmed 0.1 M phosphate buffer containing 2 mM MgCl_2_, 0.2% NP-40, and 0.1% sodium deoxycholate for 10 min at 37 °C before being transferred to a solution of the same composition plus 5 mM ferrocyanide (Sigma, P3289), 5 mM ferricyanide (Sigma, 455946), and 1.5 mM of 5-bromo-4-chloro-3-indolyl-β-D-galactoside (X-Gal, 5Prime, 2500040) for 90 min. The reaction was stopped by washing 3 times at room temperature with 1x HEPES buffered saline containing 0.1% Triton X-100 and 1 mM EDTA. Sections were then fixed for 1 h at 4 °C in 4% PFA/PBS and washed several times in PBS before being counterstained as described below.

After stopping the β-galactosidase stain, the slides were defatted by dehydration into xylene and then rehydrated in tap water. Slides were counterstained for 1 min at room temperature with a 1-to-3 dilution of nuclear fast red stock solution (0.1% nuclear fast red Kernechtrot; Fluka, 60700) and 5% aluminum sulfate hydrate (Mallinckrodt, 3208) until the sections attained a light pink color. The slides were then rinsed in running tap water and dehydrated into xylene before being coverslipped with Permount (Fischer Scientific, S70104). Sections from Prnp-tTA mice were counterstained with Neutral red, while the sections from Camk2a-tTA mice were counterstained with Cresyl Violet, as described in the original publications^4,21^.

### Immunohistochemistry for validation of Pitx3 and Pcp2 staining

To validate the cellular identity of X-gal positive cells in the midbrain of Pitx3-tTA and cerebellum of Pcp2-tTA animals, a limited selection of sections adjacent to those used in the primary X-gal analysis from Pitx3-tTA (Mouse 6513) and Pcp2-tTA (Mouse 4340) animals were co-immunostained for β-galactosidase alongside specific markers for tyrosine hydroxylase or calbindin (see below). Prior to immunostaining, brain tissue from each section was carefully separated from the gelatin matrix with iridectomy scissors before being washed in TBS. Sections were blocked for 1 h at room temperature with TBS containing 0.1% Triton-X and 5% normal goat serum before being incubated overnight at 4 °C in blocking solution containing chicken anti-GFP (1:500, Abcam, ab13970) and either rabbit anti-tyrosine hydroxylase (TH; 1:500, Millipore, AB152) or mouse anti-calbindin D-28K (1:2,000, Swant, McAB 300). Sections were washed before incubation in secondary antibody diluted in blocking solution (Alexa 488-conjugated goat anti-chicken; 1:500, Invitrogen, A-11039) and either Alexa 568-conjugated goat anti-rabbit (1:500, Invitrogen, A-11011) or Alexa 568-conjugated goat anti mouse (1:500, Invitrogen, A-11031). Sections were then washed, mounted, and coverslipped using Vectashield mounting medium containing DAPI (4′,6-diamidino-2-phenylindole) counterstain (Vector Laboratories, Burlingame, CA, USA).

### Brightfield microscopic imaging

Brightfield microscopy images from Camk2a-tTA^21^ and Prnp-tTA^4^ mice were acquired using an automated Olympus Bx52 microscope, equipped with a motorized stage (LEP MAC5000, LUDL Electronic Products Ltd., Hawthorne, NY, USA), an Optronics MicroFire digital camera (Optronics Goleta, CA USA), and Neurolucida v6.0 Virtual Slice software (MicroBrightField Inc., Williston, VT, USA) with a 20x objective yielding images with a resolution of 1.4 pixels/μm. All other images were acquired using a Zeiss Axioscan Z1 slide scanner running Zeiss Zen Software (Carl Zeiss MicroImaging, Jena, Germany) with a 20x objective yielding images with a 0.22 μm/pixel resolution. Images were exported in Tagged Information File Format (TIFF).

### Fluorescence microscopic imaging

Fluorescence microscopy images of selected immunostained sections from one Pitx3-tTA and one Pcp2-tTA animal (see above) were imaged with a Zeiss Pascal laser scanning microscope (Zeiss, Germany) using a Plan Neofluar, 40x immersion objective (n.a. 1.3, oil). Images were acquired sequentially, with a pinhole size of about 1 airy unit, and a scan speed of 12–13 μs/pixel. Laser wavelengths for the green and red channels were 488 and 543 nm, respectively. The Z-stacks were obtained according to the Nyquist theorem with an optical slice of 0.9 μm, using a slice thickness of 0.45 μm. Excited with the appropriate laser, the fluorophores were captured sequentially, i.e. in green (goat anti-chicken 488) and red (goat anti-rabbit 568).

### Registration with anatomical reference atlas

**Figure 2:**
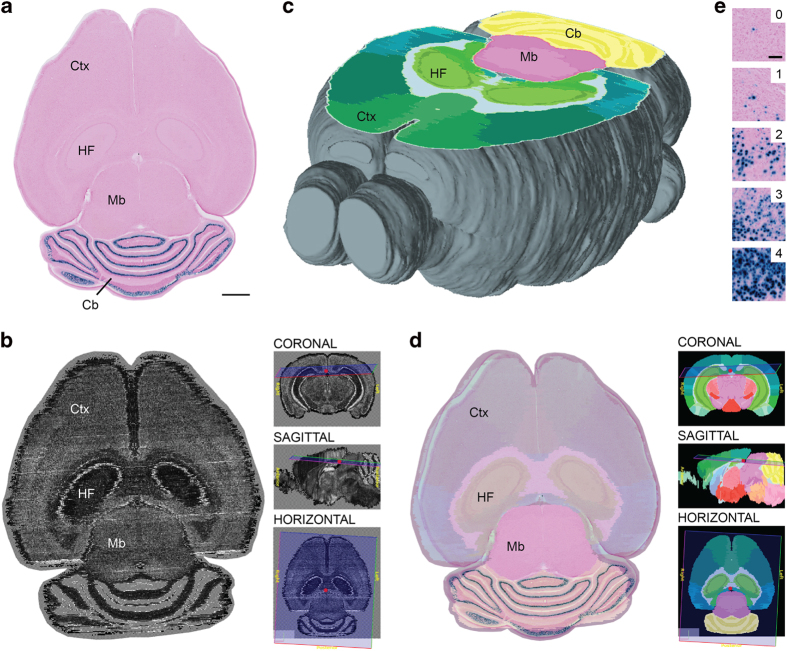
Registration of images to the Allen Mouse CCF v.2 reference atlas. Illustration of the principle used for spatial registration of microscopic mouse brain images to a three-dimensional reference atlas (**a–d**) and semi-quantitative scoring system used for analysis of labeling (**e**). (**a**) Image of a X-gal labeled horizontal section from the brain of a Pcp2-tTA mouse. (**b**) Slice from the Allen Mouse CCF v.2 reference atlas with position and angle of orientation matching the experimental section in (**b**). Insets indicate the virtual sectioning plane in coronal, sagittal, and horizontal views. (**c**) 3-D rendering of the Allen Mouse CCF v.2 atlas showing the virtual cutting plane used in (**b**) with delineations of major brain structures. This custom atlas diagram is superimposed and spatially aligned with the original section image (**d**). (**e**) A semi-quantitative grading scale from 0 to 4 is used to score the density of labeled elements in different brain regions (see methods section for details). Cb, cerebellum; Ctx, cerebral cortex; HF, hippocampal formation; Mb, midbrain. Scale bars, (a–e) 1 mm (e) 100 μm.

**Figure 3:**
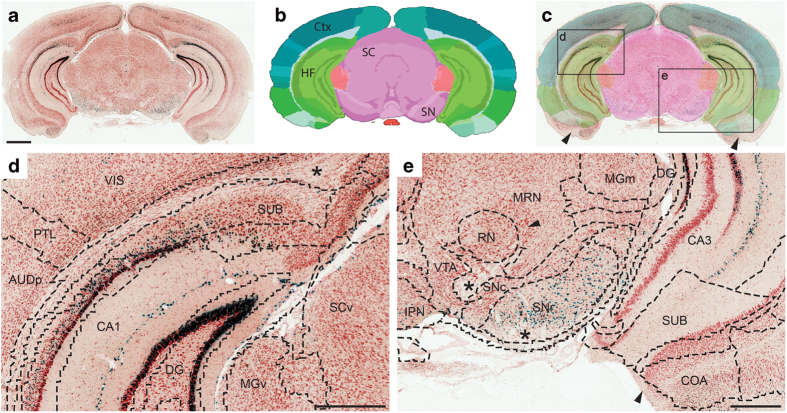
Precision of image registration to atlas. (**a**) X-gal stained coronal section from a Prnp-tTA/tetO-lacZ mouse (case #388.12). (**b**) A corresponding custom virtual atlas map from the Allen Mouse CCF v.2. (**c**) Semi-transparent atlas map superimposed on the section image. (**d**,**e**) Higher magnifications from framed areas indicated in (**c**), with dashed lines indicating anatomical boundaries derived from the atlas. Note the spatial correspondence between atlas delineations and the underlying cytoarchitecture in the hippocampal region (see, e.g. CA1, CA3, DG, SUB) and midbrain (e.g. SNc, SNr), and the partial mismatch of the atlas delineations with the ventral part of the brain (arrowheads in **c,e**). CA1, cornu ammonis area 1; CA3, cornu ammonis area 3; cc, corpus callosum; Ctx, cerebral cortex; COA, cortical amygdalar area; DG, dentate gyrus ; GENd, medial geniculate complex; HF, hippocampal formation; MB, midbrain; MRN, midbrain reticular nucleus; PA, posterior amygdalar nucleus; PAG, periaqueductal gray; RN, red nucleus; PP, peripeduncular nucleus; RSP, retrosplenial area; SC, superior colliculus; SNc, substantia nigra compact part; SNr substantia nigra reticular part; SPF, subparafascicular nucleus; SUB, subiculum; VIS, visual cortex; VTA, ventral tegmental area. Scale bar, 1 mm.

All images were registered to the Allen Mouse CCF v.2^24^ using the QuickNII software tool^32^. This semiautomatic tool provides custom atlas maps cut at angles of orientation matching the experimental images, which can compensate for deviations in data collected from standard cutting planes. The atlas maps are superimposed and registered to the experimental section images (Fig. 2) by use of affine transformations (scaling, panning, and rotation) to achieve a global match of multiple anatomical landmarks (Fig. 3). For the semi-quantitative analysis (see below), the number of atlas structure delineations was reduced by grouping regions that were smaller than ~200 μm in diameter.

### Web-based interactive display of images

For evaluation of β-galactosidase labeling, images were organized in the Navigator3 web-based image management system developed and hosted by the University of Oslo, Norway. This system provides access to an interactive web-microscopy viewer tool that allows users to display microscopic images with atlas overlay atlas maps, annotate points of interest in reference atlas space, and view annotated points of interest together with three-dimensional (3-D) surface models of the Allen Mouse Brain Atlas.

### Semi-quantitative scoring of labeled cells

**Figure 4:**
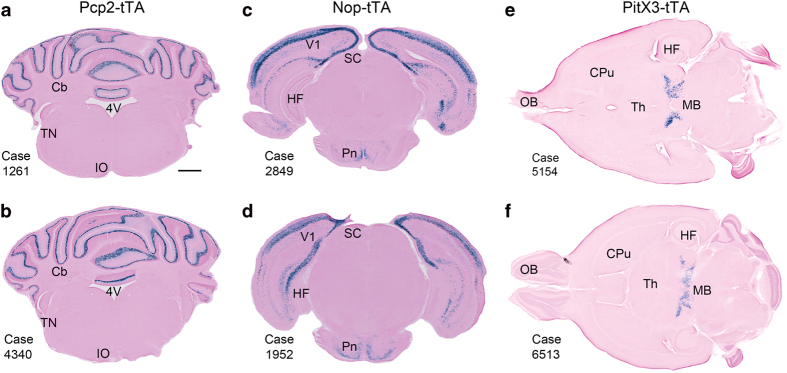
Consistency of labeling across animals. Images of spatially corresponding sections from Pcp2-tTA/tetO-lacZ-nls-GFP (**a**,**b**), Nop-tTA/tetO-lacZ-nls-GFP (**c**,**d**) and Pitx3-tTA/tetO-lacZ-nls-GFP (**e**,**b**) animals showing highly consistent patterns of X-gal labeling in animals of the same strain. 4 V, fourth ventricle; Cb, cerebellum; CPu, caudate putamen; HF, hippocampal formation; IO, inferior olive; MB, midbrain; OB, olfactory bulb; Pn, pontine nuclei, SC, superior collicle; Th, Thalamus; TN, trigeminal nerve; V1, primary visual area. Scale bars, 1 mm.

To provide comparable measures of the X-gal-labeled cells across different brain regions in all animals (Data Citation 1), the density of labeling was assessed by two independent researchers using a semi-quantitative grading system from 0–4, introduced in Yetman *et al.*^22^ (Fig. 2e). Here grade 0 represents absence of labeled cells (less than 1 per 0.01 mm^2^), grade 1 - low density (few cells, possible to count), grade 2 - medium density (several cells that can be individually discerned, but not readily counted), grade 3 - high density (many labeled cells with large degree of overlap), and grade 4 - very high density (where individual cells cannot be discerned). The comparison is based on semiquantitive scores of labeling in one representative case for each tTA line (Data Citation 1), with all results verified in the other cases (Fig. 4). Scores did not vary more than 1 grade between cases or researchers in any regions. The highest numbers were reported. If the density of labeling was found to vary substantially within a region, the highest observed score was recorded.

### Code availability

The image data are shared in standard TIFF format that can be viewed and analyzed with a range of tools. The software used to register images to the references atlas (QuickNII) is shared via NITRC (www.nitrc.org/projects/quicknii). The software tools (LocaliZoom, MeshView) used here for visualization and analysis are embedded in the Navigator3 image management system, developed and hosted by the Neural Systems Laboratory at the University of Oslo, Norway. These tools are available from the Human Brain Project web page (www.humanbrainproject.eu) upon user registration.

### Registration and curation of data in the Human Brain Project infrastructure

Image data and metadata were made publicly available through services for data storage, management, and professional data curation offered by the EU Human Brain Project (www.humanbrainproject.eu). The data are Findable, Accessible, Interoperable, and Reusable, in accordance with the FAIR guiding principles^33^. The Human Brain Project curation service provides DataCite Digital Object Identifyers (DOIs) for each data set, structured metadata describing basic parameters on data provenance, compliance with regulations on ethical conduct, conditions for use, license for sharing, and validated location metadata defining the location of data in a common anatomical reference atlas.

## Data Records

The image files and associated metadata are shared via the web page of the Human Brain Project (www.humanbrainproject.eu; Data Citation 2, 3, 4, 5, 6, 7, 8, 9, 10, 11, 12, 13). The tTA image collection comprises series of microscopic images from five tTA-expressing transgenic mouse lines (n = 1–4 animals/line). Each data set consists of series of uncompressed high-resolution TIFF images and a data descriptor file (TXT format) and has been given a DataCite DOI. For each data set, a link is provided to a web-microscopy viewer tool featuring functions for exploring and analyzing data, including 1) interactive zooming and panning of images, 2) toggling of customized atlas delineations registered to each image, 3) hover-activated display of anatomical names, and 4) annotation and exact spatial coordinates for points of interest defined in the Allen Mouse CCF v.2 reference atlas. The comparative overview of labeling density for each of the tTA lines is provided as a derived data set with a separate DOI (Data Citation 1). Table 1 provides an overview of the materials included. The ‘usage notes’ below provide suggestions about how the interactive web-microscopy viewer can be engaged.

The images are also shared in the Navigator3 image management system accessible from the Rodent Brain Workbench (www.rbwb.org; select “Brain atlas of tTA driver lines”). This web resource gives 1) overview of the data sets included in the present collection, 2) links to the Human Brain Project web-microscopy viewer system, and 3) options for downloading collections of original TIFF images with spatially matching low resolution Portable Network Graphics (PNG) atlas maps.

All image series include coronal brain sections, with horizontal sections available for the Nop, Pitx3, and Pcp2 driver lines (Table 1; Data Citation 2, 3, 4, 5, 6, 7, 8, 9, 10, 11, 12, 13). Each series contains between 15 and 57 images, sampled to cover all major brain regions. The file names encode the animal number (first four digits), genotype, staining (X-gal), serial number (_s followed by three digits), and pixel dimensions (extension _1.4 or _0.22, indicating μm/pixel). For each microscopic image, an atlas map is provided as a 1024 pixel-wide PNG image that represents the same physical space as the higher resolution microscopic images. Each atlas map is named to match its corresponding TIFF image with an additional extension (_segmentation). The total folder size for each series ranges between 4 and 11 gigabytes.

The microscopic images of coronal sections range in size from 25,000 × 30,000 pixels to 35,000–55,000 pixels (file size 42–230 megabytes, LZW compressed TIFF), while horizontal images range in size from 35,000 × 35,000 to 50,000 × 70,000 pixels (file size 100–350 megabytes, LZW compressed TIFF). It should be noted that several of the original TIFF files exceed the maximum limit that can be opened in some common image processing software. These images can, however, be viewed using open-access software tools like e.g. GIMP (https://www.gimp.org/).

## Technical Validation

Data quality was ensured for each step of data collection and processing. The histological material was prepared to preserve morphological integrity and to cover all major brain regions. Counterstaining was used to highlight anatomical boundaries. Care was taken to define the orientation of section planes, and to ensure proper serial order of the sections. All section images were registered to a common mouse brain reference atlas. This facilitates comparison across specimens, even when the plane of sectioning differs. The accuracy of registration was validated by two independent researchers. The density of cellular labeling was scored by two independent researchers. Consistency of labeling was validated by analysis of X-gal labeling in multiple animals from each line.

### Sensitivity and specificity of X-gal staining

X-gal staining is based on the enzymatic cleavage of a colorimetric substrate that continues until the reactants are fully depleted. This stain thus provides a sensitive readout of β-galactosidase localization, but does not offer a quantitative measure of tTA expression levels. Since lysosomal β-galactosidase is present in all tissues, and some tissues have endogenous enzyme activity^34^, background labeling should be evaluated and excluded. For three of the five tTA lines examined (Nop, Pitx3, and Pcp2), this could be done by directly comparing the overlap between X-gal labeling and GFP fluorescence co-expressed in the reporter construct. The GFP signal was overall less intense compared to X-gal, but the expression pattern largely overlapped. Additionally, no X-gal labeling could be detected in single transgenic control animals (tetO-lacZ-nls-GFP) stained alongside the bigenic samples^22^.

Since the same protocol was used to visualize X-gal staining in all of the models, the semi-quantitative assessment of labeling density should be comparable across lines. It should be noted, however, that tissue sections differ in thickness and were processed in different laboratories.

### Variability in ages and sexes

The present repository provides the first comprehensive comparison of brain-wide expression patterns in tTA driver-reporter lines. It furthermore demonstrates that it is possible to build repositories of comparable image data originating from different laboratories. Both male and female mice are included in the present data collection and animals span a range of ages from 5–42 weeks at harvest. (Table 1). While expression patterns in these lines are not known to vary with age or sex in adulthood, this possibility cannot be excluded and should be taken into consideration when interpreting the data. Since the data repository is hosted in an infrastructure allowing addition of data sets, the present collection can be expanded with future data sets that may clarify whether expression varies in any of the lines as a function of age or sex.

### Accuracy of image registration

The registration of custom atlas maps to each section image is achieved by considering multiple anatomical landmarks for each section image, as well as for its adjacent images. The approach used here was to match the atlas map globally to the image, i.e. most landmarks will have a correct position. The anatomical accuracy of image registration to the common reference atlas was validated by two independent researchers. Overall, most brain regions aligned well with the atlas (Fig. 3), but registration was less precise in some brain regions that were displaced or distorted during histological processing, or different in shape relative to the atlas used (Fig. 3c,e). Such registration errors are difficult to quantify, but may vary from tens to hundreds of micrometers. Comparison of 3-D distributions of labelled cells between four cases sectioned in different planes of orientation (see below), confirms that the registration of images to the reference atlas was sufficiently accurate to yield consistent results across cases. Nevertheless, in regions where differences in shape could not be compensated by affine transformations, the position of the atlas overlays were temporarily adjusted during the semi-quantitative evaluation of expression density, to achieve a better match between local anatomical landmarks and ensure appropriate structural assignment.

### Validation of the morphological phenotype of Pitx3 and Pcp2 lines

Pitx3 is a transcriptional regulator important for differentiation and maintenance of meso-diencephalic dopaminergic neurons during development, and the Pitx3-tTA line was created to investigate degeneration of these cells in Parkinson’s disease^19^. Purkinje cell protein 2 (Pcp2) is expressed in cerebellar Purkinje cells and retinal bipolar cells, and the Pcp2-tTA line was created to investigate neural degeneration in a mouse model of spinocerebellar ataxia type 1^35^. In these lines, the cellular identity of X-gal-positive cells was confirmed by immunostaining against tyrosine hydroxylase for dopaminergic cells or calbindin for Purkinje cells.

**Figure 5:**
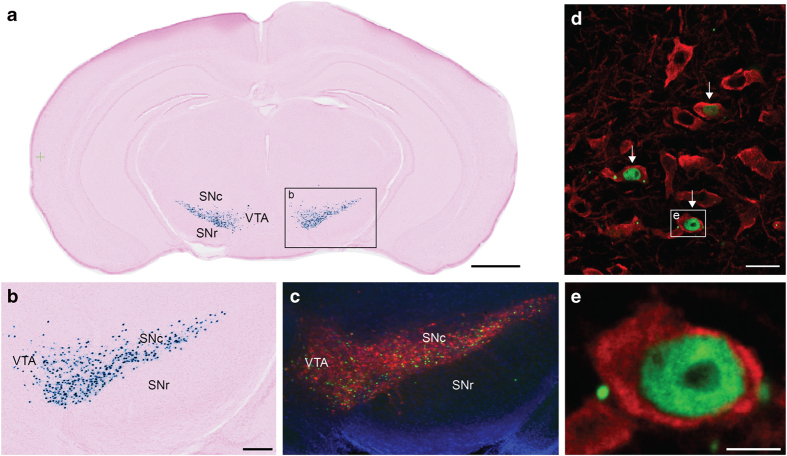
Validation of the cell-type specificity in the Pitx3-tTA line. (**a**) X-gal stained coronal section from a Pitx3-tTA/tetO-lacZ-nls-GFP mouse brain, with atlas derived annotations shown in the left hemisphere. (**b**) Enlarged detail from (**a**) showing part of the midbrain containing the substantia nigra. (**c–e**) Confocal microscopic images with increasing magnification of a green fluorescent protein (GFP), tyrosine hydroxylase (TH) and DAPI stained section adjacent to the section in (**a**,**b**). TH immunohistochemistry is used to identify dopaminergic cells in the brain, and TH positive cells appear red in (**c–e**). Cells co-labeled with GFP and TH (arrows) in the SNc are shown in (**d**) and a single co-labeled cell in (**e**). Note that GFP, and thus also lacZ, is expressed only in a subset of TH positive cells. SNc, substantia nigra compact part; SNr, substantia nigra reticular part; VTA, ventral tegmental area. Scale bars, (a) 1 mm (b,c) 100 μm (d) 20 μm (e), and 2 μm.

**Figure 6:**
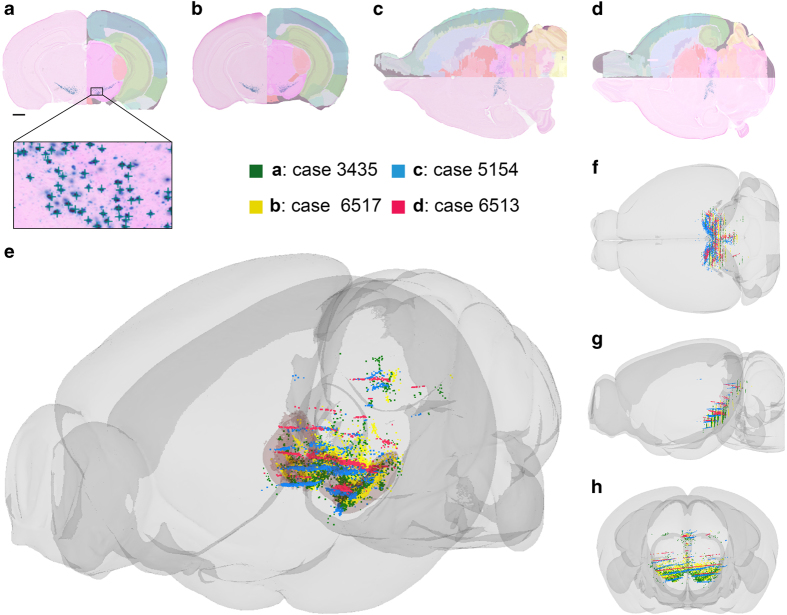
3-D representation of data from different Pitx3-tTA cases. (**a–d**) Section images showing X-gal labeling in four Pitx3-tTA cases, two sectioned coronally (**a**,**b**) and two sectioned horizontally (**c,d**), with atlas overlay shown on one side. The positions of labeled cells were manually recorded from all section images by placing point coordinates on each labeled cell using the annotation tool in the LocaliZoom viewer (exemplified in the enlarged inset in **a**). (**e–h**) The point coordinates were color-coded and co-visualized together with atlas structures in a 3-D viewer. Views from anterolateral (**e**), dorsal (**f**), lateral (**g**), and anterior (**h**) are provided. In (**e**), the outer boundaries of the substantia nigra and ventral tegmental area are shown as transparent pink surfaces. The 3-D visualization allows comparison of data derived from coronal and horizontal section images, and demonstrates that labeled cells are similarly distributed in the four Pitx3-tTA cases, with high densities in the substantia nigra and ventral tegmental area, but also with labeling in more dorsal parts of the mesencephalon. Scale bar, 1 mm.

In sections from Pitx3-tTA brains, dense populations of X-gal labeled cells were found in the substantia nigra pars compacta (SNc) and the ventral tegmental area (VTA), corresponding well with earlier publications (Fig. 4e,f and Fig. 5a,b)^19,36^. Confocal microscopic examination of sections immunostained for tyrosine hydroxylase confirmed earlier reports that the expression of Pitx3-tTA was restricted to a subset of dopaminergic neurons in the SNc and VTA (Fig. 5c–e). Some X-gal labeling was also observed in the midbrain raphe nuclei, inferior colliculus, midbrain reticular nucleus, and periaqueductal grey, indicating some Pitx3-tTA promoter activity also occurs outside the SNc and VTA. This is illustrated in Fig. 6, showing a 3-D co-visualization of the location of labeled cells. In two of the four animals investigated, a few scattered X-gal labeled cells were also found in the main and accessory olfactory bulb.

**Figure 7:**
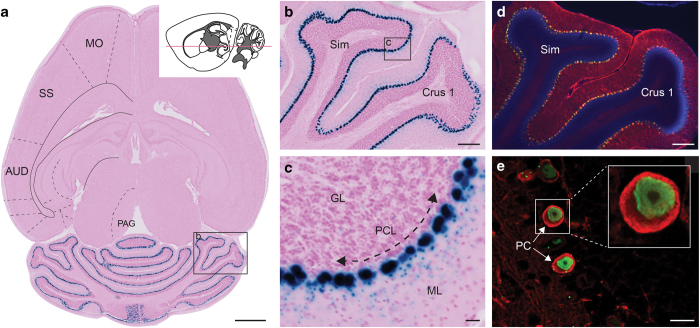
Validation of cell-type specificity in the Pcp2-tTA line. (**a**) X-gal stained horizontal section from a Pcp2-tTA/tetO-lacZ-nls-GFP mouse brain. The left hemisphere has annotations corresponding to a virtual atlas map from the Allen Mouse CCF v.2. The cartoon drawing of a sagittal section shows the approximate cutting plane as a red line. (**b**) Enlarged detail from the section in (**a**) showing part of the cerebellum. (**c**) Further magnified detail from the section in (**a**,**b**) showing labeling between the cerebellar granular layer and the molecular layer. (**d**) Confocal microscopic image of a green fluorescent protein (GFP), calbindin, and DAPI stained section adjacent to the section in (**a–c**). Calbindin is a marker for Purkinje cells, and with high magnification one can clearly identify cells co-labeled for GFP and calbindin (**e**). Since expression of GFP and lacZ are both driven by the Pcp2 promoter, this verifies that the X-gal labeled cells in (**a–c**) are also Purkinje cells. AUD, primary auditory area; Crus 1, cerebellar crus 1, GL, granular cell layer; ML, molecular cell layer; MO, primary motor area; PAG, periaqueductal gray; PC, Purkinje cell; PCL, Purkinje cell layer; SS, primary somatosensory area; Sim, simple lobule. Scale bars, (a) 1 mm (b,d), and 200 μm (c,e) 20 μm.

In sections from Pcp2-tTA brains, X-gal labeling was exclusively observed in cerebellar Purkinje cells (Fig. 4a,b and Fig. 7a–c), consistent with previous reports^35,37^. Confocal microscopy of sections immunostained for calbindin confirmed that tTA dependent expression was restricted to Purkinje cells (Fig. 7d,e). The labeled cells were distributed across the entire cerebellum, including most, but not all Purkinje cells.

## Usage Notes

### Accessing and viewing images from tTA driver lines

Each data set can be directly accessed via DOIs (Data Citation 2, 3, 4, 5, 6, 7, 8, 9, 10, 11, 12, 13), or query tools provided via the Human Brain Project web page. For each data set, a landing page provides access to downloadable high-resolution image files, basic metadata with data provenance and license information, and a link to an interactive viewer tool. The viewer provides a “filmstrip” overview of serial images from one animal, and features panning and zooming of individual high-resolution images, with a semi-transparent graphical overlay images showing the custom atlas mapped to fit the images. The atlas diagrams provide a reasonable starting point for anatomical analyses, but additional methods may be necessary to make more precise statements about anatomical locations.

### Examples of use

Below, we outline three example scenarios in which the present collection of images from tTA driver lines may provide a useful resource.

#### Example 1: Comparing tTA expression patterns to find a driver line suitable for conditional transgene expression in a specific region of interest

**Figure 8:**
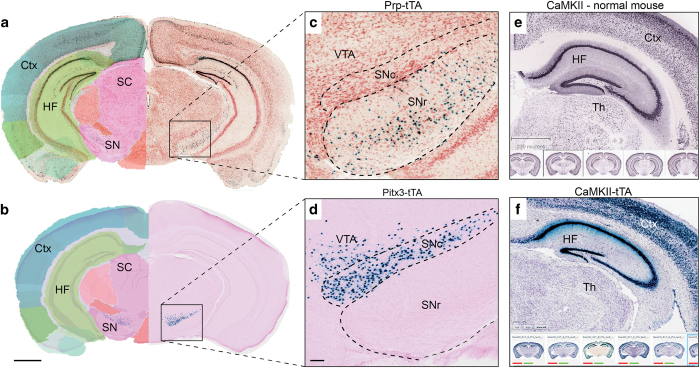
Examples of use. (**a–d**) X-gal labeled coronal sections through the midbrain from Prnp-tTA/tetO-lacZ (**a**,**c**) and Pitx3-tTA/tetO-lacZ-nls-GFP (**b**,**d**) mice. The left hemispheres are presented with semi-transparent overlays of custom atlas maps as available in the online viewer. (**c**,**d**) Higher-magnification details from the substantia nigra showing notably different labeling patterns between the two lines. In this region the Prnp-tTA line mainly has labeled cells within the reticular part (**c**), while the Pitx3-tTA line displays labeling highly restricted to the compact part and the ventral tegmental area. (**d**). Note that for the Prnp-tTA line, labeling also occurs in several other regions such as the hippocampal formation and the cerebral cortex. (**e**) Shows a screenshot of *in situ* hybridization data representing gene distribution of the Camk2a gene in the hippocampal area of a normal mouse from the Allen Institute repository (www-brain-map.org). (**f**) Shows a screenshot of a corresponding X-gal labeled section from a Camk2a-tTA/tetO-lacZ mouse, available from the rodent brain workbench. Similar viewers facilitate comparison of native gene distribution patterns and tTA dependent transgene expression patterns. Ctx, cerebral cortex; HF, hippocampal formation; SC, superior colliculus; SN, substantia nigra; SNc, substantia nigra, compact part; SNr, substantia nigra, reticular part; Th, thalamus; VTA, ventral tegmental area. Scale bars, 1 mm (a,b), 100 μm (c,d).

A researcher investigating Parkinson’s disease wants to create a conditional mouse model in which she can specifically regulate the expression of a gene of interest in the substantia nigra. She chooses to use the tet-transactivator system, and therefore needs a tTA-expressing line active in her region of interest. She queries the Human Brain Project database for tTA driver lines, and finds several collections of image data and a derived data set providing an overview of promoter expression (Data Citation 1). The tabular overview shows that two tTA driver lines are active in the substantia nigra. The Prnp-tTA line features widespread X-gal labeling in numerous brain regions, including the substantia nigra, while the Pitx3-tTA line has more spatially restricted labeling in the substantia nigra, ventral tegmental area, and a few other regions. She clicks on the link to launch the online virtual microscope viewer, in which she can inspect and compare serial images and corresponding atlas maps from these two lines (Fig. 8), or download original images for use with other tools. Since sections from both lines are registered to the same reference atlas, she can easily find and compare corresponding sections. Using the online image viewer she can also identify the coordinates of a point of interest in one section and compare this to coordinates extracted from other image series (Fig. 6). In this way she can quickly examine the spatial pattern and specificity of reporter expression in the two relevant driver lines, and consider whether this is compatible with her desired experimental paradigm. Inspecting corresponding sections from the two lines, she discovers that within the substantia nigra, the Prnp promoter line mainly drives transgene expression in the reticular part, while Pitx3 promoter activity is restricted to the compact part (Fig. 8a–d). For the Pitx3-tTA line she can look up additional documentation confirming that the labeled cells are positive for tyrosine hydroxylase and therefore dopaminergic neurons. The Prnp-tTA line drives transgene expression in the reticular part of the substantia nigra (Fig. 8a,c), but also in many other brain regions (Fig. 3a), while the Pitx3-tTA line drives transgene expression selectively in dopaminergic neurons of substantia nigra pars compacta and ventral tegmental area (Fig. 8b,d). With this information, she can choose the tTA line best suited for her purposes. Finally, to compare the spatial distribution of labeling among cases from one tTA line, she can also use the manual annotation function in the online image viewer tool to record the positions of labeled cells in the reference atlas space for all sections from the Pitx3-tTA cases, and co-display these in a 3-D viewer tool (Fig. 6).

#### Example 2: Interpreting findings from a disease model using a tTA driver line

The Nop-tTA driver line has been described as primarily restricted to the medial entorhinal cortex and pre- and parasubiculum^29^, but brain-wide examination of reporter expression has revealed activity in many additional brain regions^22^. A researcher investigating possible axonal spread of pathological protein aggregates in a conditional mouse model of Alzheimer’s disease based on the Nop-tTA line would like to know which cells initially express the transgenic protein of interest. The researcher expects pathological aggregates will appear in regions where the responder gene is transcribed and possibly also in regions to which these cells project axonal connections. The interpretation of findings thus requires specific knowledge of where the Nop-tTA driver is active and the axonal connections of those regions. The researcher can use the tabular overview of the online tTA atlas to identify where X-gal labeling occurs in Nop-tTA mice (Data Citation 1). Subsequently he can inspect the detailed labeling patterns in coronal (Fig. 4c,d) and horizontal sections. He can then enlist other resources such as the Allen Mouse Brain Connectivity Atlas to examine neural connections made by the identified regions^24^. The interactive Connectivity Atlas provides access to a large number of volumetric images showing anterograde axonal labeling across entire mouse brains. Using the tabular overview of brain regions with X-gal labeling in Nop-tTA mice as a starting point, the researcher can systematically cross-check the outcome of tracer injections to corresponding locations in the Allen Mouse Brain Connectivity atlas (http://connectivity.brain-map.org). Such comparisons are readily done since all images in the Brain atlas of tTA driver lines are spatially defined using the common reference atlas.

#### Example 3: Comparing transgene expression in tTA driver lines with gene distribution patterns from other repositories

A researcher studying gene expression in the mouse brain wants to compare the spatial distribution of promoter activity in a tTA transgenic line with that of a particular transcript in the normal mouse brain. The Allen Institute hosts data from *in situ* hybridization experiments showing gene expression in the adult mouse brain for > 20,000 independent transcripts (http://mouse.brain-map.org). The Allen Institute repository can be queried for genes of interest, and expression patterns can be viewed in serial section images. The researcher can then perform side-by-side comparisons of corresponding sections from the Brain atlas of tTA driver lines and the Allen Institute repository (Fig. 8e,f), or download images for more detailed analysis using other tools.

### Future use and expansion of the image repository

All images shared in the present data collection have been spatially integrated by registration to a common reference atlas and made publicly available. This facilitates integration of data not only within the collection, but also makes it possible to relate our tTA data with the growing collection of heterogeneous data accumulated by the Human Brain Project. The Human Brain Project database is open for new data, and provides data curation services and tools for registering image data to the Allen Mouse Common Coordinate Framework^24^. The present collection of tTA image data is now available for new data to be added: should other investigators conduct similar intercrosses on new driver-reporter lines, their microscopic image data can leverage the workflow developed here to integrate new data sets with the existing collections. Inclusion of data from additional tTA lines or from other ages/sexes of the tTA lines presented here will substantially increase the scientific value of the repository. Researchers working with tTA animals are therefore encouraged to share their data and expand this public collection of tTA promoter expression data.

## Additional information

**How to cite this article**: Lillehaug, S. *et al*. Brain-wide distribution of reporter expression in five transgenic tetracycline-transactivator mouse lines. *Sci. Data*. 6:190028 https://doi.org/10.1038/sdata.2019.28 (2019).

**Publisher’s note**: Springer Nature remains neutral with regard to jurisdictional claims in published maps and institutional affiliations.

## Supplementary Material



## Figures and Tables

**Table 1:** Overview of materials.

	Driver line/Reporter line	Animal #	Gender	Age (weeks)	# Images	Orientation
Camk2a	Camk2a-tTA/tetO-lacZ	317.8	female	41	47	coronal
Nop	Nop-tTA/tetO-lacZ-nls-GFP	1952	male	38,5	46	coronal
	Nop-tTA/tetO-lacZ-nls-GFP	2877	male	36	23	horizontal
	Nop-tTA/tetO-lacZ-nls-GFP	2849	female	31	34	coronal
Pcp2	Pcp2-tTA/tetO-lacZ-nls-GFP	1261	male	42,5	41	coronal
	Pcp2-tTA/tetO-lacZ-nls-GFP	3292	female	37	23	horizontal
	Pcp2-tTA/tetO-lacZ-nls-GFP	4340	male	20	33	coronal
Pitx3	Pitx3-tTA/tetO-lacZ-nls-GFP	3435	male	15	42	coronal
	Pitx3-tTA/tetO-lacZ-nls-GFP	5154	female	12,5	16	horizontal
	Pitx3-tTA/tetO-lacZ-nls-GFP	6513	male	9	15	horizontal
	Pitx3-tTA/tetO-lacZ-nls-GFP	6517	female	9	32	coronal
Prnp	Prnp-tTA/tetO-lacZ	388.12	female	5	57	coronal
